# Regulatory T Cells in Development and Prediction of Necrotizing Enterocolitis in Preterm Neonates: A Scoping Review

**DOI:** 10.3390/ijms231810903

**Published:** 2022-09-18

**Authors:** Mara O. Zuiderwijk, Mirjam van der Burg, Vincent Bekker, Michiel H. D. Schoenaker

**Affiliations:** 1 Willem Alexander Children’s Hospital, Division of Neonatology, Department of Pediatrics, Leiden University Medical Center, 2333 ZA Leiden, The Netherlands; 2 Willem Alexander Children’s Hospital, Laboratory for Pediatric Immunology, Department of Pediatrics, Leiden University Medical Center, 2333 ZA Leiden, The Netherlands

**Keywords:** necrotizing enterocolitis, NEC, preterm, regulatory T cell, Th17 cell

## Abstract

Necrotizing enterocolitis (NEC) is a leading cause of mortality in premature infants. However, the pathophysiology and influence of regulatory T cells (Tregs) have not been sufficiently elucidated. We performed a scoping review to investigate current knowledge on the influence of Tregs in NEC, and to investigate the predictive value of Treg number in NEC development. Pubmed, Embase, Prospero and Cochrane Library were searched during December 2020. Primary research articles discussing Tregs and NEC development written in English were selected. Two reviewers screened title and abstract for relevance, after which full-text screening was performed. A total of 20 articles were selected—13 of the articles discussed studies performed in animal models, while 8 used human neonate data. One study discussed both animal and human data. It was shown that after NEC diagnosis or induction, Treg levels were decreased while Th17 levels were increased. No studies were found which investigated the predictive value of Treg number in NEC development. A reduced Treg level is found in animals and neonates with NEC. The question remains whether this effect is a factor on the causal pathway of NEC development or a bystander effect. Future research focusing on the pathophysiological timeline of NEC and the involvement of Tregs is required for better understanding of this disease.

## 1. Introduction

Necrotizing enterocolitis (NEC) is a life-threatening disease, characterized by inflammation and necrosis of the intestinal wall in premature infants. NEC is considered a leading cause of morbidity and mortality in the neonatal intensive care unit (NICU), as approximately 20–30% of affected children succumb [1]. Those who survive face increased risk of other complications including difficulties in feeding, delayed development, late onset sepsis, short bowel syndrome and severe neurodevelopmental impairment [2]. Current management is largely non-specific, including broad-spectrum antibiotics, bowel rest and fluid provision, and maintenance of cardiorespiratory function [1]. Surgical removal of necrotic intestine is required in up to 50% of neonates with severe NEC [3].

Despite the complex and multifactorial nature of the pathogenesis of NEC, three major risk factors have been implicated in its development: prematurity, bacterial colonization of the gut and formula-feeding.

The direct causes of NEC remain unclear, but may arise on the basis of the interactions between two poorly developed systems, namely the intestine and the immune system [4]. Immaturity of the intestinal mucosa and reduced barrier functions could lead to bacterial translocation. The inflammation overwhelms the counter-regulatory mechanisms, and in turn leads to cell death. As a result, intracellular components such as stored cytokines are released, leading to an inflammatory storm and initiation of the cascade which ultimately leads to NEC [4].

Previous research showed that the number of regulatory T cells (Tregs), an important source of anti-inflammatory cytokines, was reduced in the ileum of NEC-rats and human infants with NEC compared to controls [5,6]. This indicates that while Tregs are present in the intestines, their numbers might be insufficient to dampen the excessive inflammatory state in NEC [7]. This inflammatory state is likely influenced by Th17 cells, a cell type which has similar cellular origins as Tregs [8]. These two cell types are able to influence each other’s differentiation by producing cytokines such as IL-6 in the case of the Th17 cells, which inhibit Treg differentiation but induce Th17 cell differentiation, and with TGFβ being required for both Treg and Th17 differentiation [9,10]. A more recent study showed that Tregs from infants with NEC displayed a multitude of functional impairments, limiting their capacity to contain the excessive inflammation-induced damage [11]. However, as samples were collected from infants after displaying NEC-like symptoms, it is unclear whether Treg impairment was a pre-existing event or a consequence of the disease. Therefore, our main research questions are whether an abnormal regulatory T cell count is predictive for the development of NEC, and how the regulatory T cell plays a role in the pathogenesis of NEC. We hypothesize that a lower level of Tregs is present in neonates with NEC compared to healthy neonates, and that in NEC development, these reduced levels cause an increased inflammatory state, which negatively influences pathophysiology. Therefore, the aim of this study was to review the literature reporting on the mechanism of regulatory T cells in experimental and neonatal NEC and to investigate whether an abnormal regulatory T cell count could be predictive for the development of NEC.

## 2. Methods

We performed a scoping review based on the PRISMA Extension for Scoping Reviews, developed by Tricco et al. [12]. The scoping question is “What is the mechanism in which the regulatory T cell plays a role in the pathogenesis of necrotizing enterocolitis?” We searched for papers using a combination of the terms “necrotizing enterocolitis” AND “regulatory T cells” in PubMed, EMBASE, Prospero and Cochrane. Additionally, for our question “Is an abnormal regulatory T cell count predictive for the development of NEC”, we added the term “predictive”. This additional term, however, resulted in zero retrieved articles; therefore, we decided to use the previously identified articles to also answer our predictive question. The search question can be found in supplemental document 1. The identified articles were evaluated using the following inclusion criteria: English language articles and primary studies of regulatory T cells in NEC. The included studies were not limited based on publication date or method. Abstracts were read by two independent reviewers, and a third reviewer had final say if no consensus was reached. If abstracts did not provide enough data to establish inclusion, the full article was reviewed. This primary screening was performed using Rayyan software (Qatar Computing Research Institute, Doha, Qatar) [13].

The protocol of this review was submitted to Prospero (CRD42021231678) prior to the start of the process.

## 3. Results

### 3.1. Study Results

**Figure 1:**
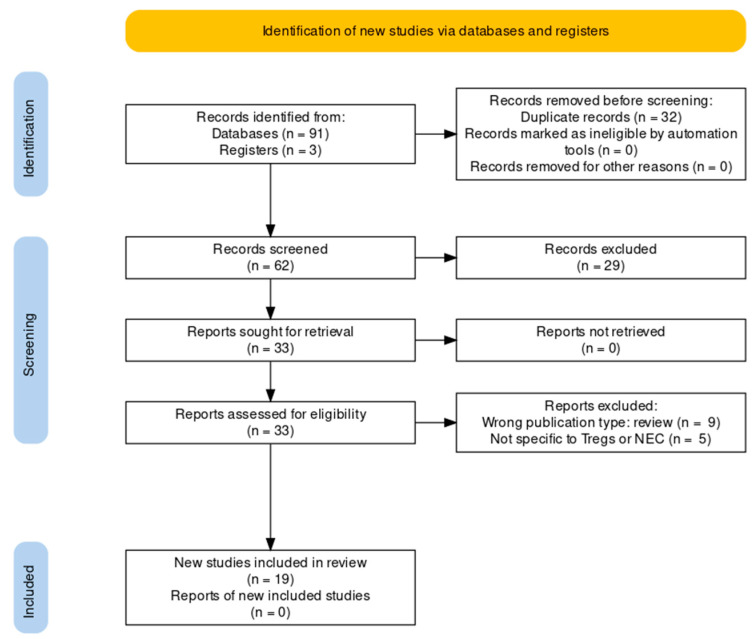
PRISMA flow chart showing locations records were identified from, abstracts from records screened, full reports assessed and total studies included [12].

In total, 62 unique articles were identified by the search strategy (Figure 1). Of these 62, 29 articles (47%) were excluded based on irrelevance of title, abstract or article type. The majority of the excluded articles were reviews, commentaries or symposium reports. The full text was retrieved for the remaining 33 articles. Of these, nine articles (15%) were excluded based on article type (reviews), and four (6%) based on the relevance of the content. The remaining 20 articles (32%) were selected for this review. A total of 65% (13) of the selected studies included experiments that were performed on animals (mice, rats, and piglets), while 40% (8) used human neonatal data and samples. One article described both animal and human data. In 79% (11) of the animal studies, NEC was induced using a protocol of formula feeding and exposing the animal to daily hypoxia for up to five days.

### 3.2. The role of Different T Cell Subsets in NEC

**Figure 2:**
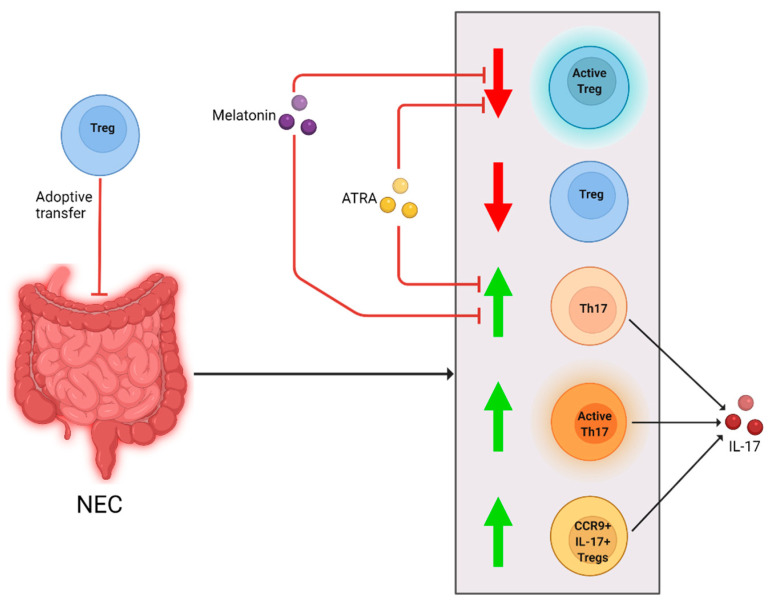
Cellular changes observed in NEC patients and animal models and different interventions. After NEC diagnosis or induction, Treg levels and activity were reduced (downward arrow). Th17 and CCR9 + IL-17+ Treg levels, and Th17 cell activity were increased (upward arrow). Melanin and ATRA negated the decrease in Tregs and increase in Th17 cells and IL-17. Adoptive Treg-transfer reduced NEC development in a mouse model.

To answer our question of whether Tregs influence NEC development, we will discuss the selected studies in which T cell ontogeny in blood or bowel tissue between NEC and non-NEC subjects are compared, or T cell differentiation and function is assessed. Findings from these articles are visualized in Figure 2 and Table 1.

#### 3.2.1. Treg Number in NEC

To start, Huenecke et al. analyzed the blood from 40 preterm infants, and 10 full-term control infants [14]. Of 40 preterm infants, 15% (6) developed NEC. In this small sample group, no significant changes in immune cell subsets were found compared to infants who did not develop NEC. This study did find that the Treg level in blood was highest in preterm infants born at a gestational age (GA) of 26 weeks compared to preterm infants born at a later GA, but declined in the first weeks of life. This was, however, not correlated to NEC development in this study, possibly because of the low sample size.

Weitkamp et al. used remnant surgical tissue of infants with NEC, perinatal spontaneous intestinal perforation (SIP) and congenital intestinal obstruction to determine ontogeny of T cells in the intestinal lamina propria [15]. In NEC remnant tissue, number of FOXP3^+^ T cells was decreased compared to non-NEC remnant tissue. FOXP3 was used as a marker for Tregs, since it is a transcription factor which induces differentiation into Treg phenotype [16]. CD3^+^, CD4^+^ and CD8^+^ T cell numbers were also decreased in NEC remnant tissue. In a second study, the group of Weitkamp et al. investigated the intestinal lamina propria of surgical patients with NEC, compared to GA-matched surgical patients without NEC [7]. In contrast to the previous study, they found that GA did not have an effect on these numbers. The ratio of Tregs to CD4^+^ or CD8^+^ T cells, however, was significantly lower in neonates with NEC compared to controls. Treg proportions increased toward control levels after NEC healing.

Dingle et al. performed a similar ileal T cell ontogeny study in rat pups after experimental NEC induction [6]. They found reduced Treg frequency in the terminal ileum of pups with experimental NEC. After Treg transfusion during NEC induction, NEC incidence and ileal damage were reduced compared to NEC induction-only rats. The transplanted Tregs were mostly found in the spleen and mesenteric lymph node, indicating that endogenous Tregs were transported to the ileum after transplantation. In rats with Treg transfer, the level of T cell activation was also reduced, leading the authors to suggest that Tregs help limit ileal T cell activation. 

In conclusion, a reduction in Treg levels was seen in preterm neonates and animal models with NEC.

#### 3.2.2. The Influence of Th17 Cells on the Inflammatory State in NEC

A similar trend in Treg frequency was reported by Egan et al. in multiple different immunological knockout mouse strains [17]. In this article, RORγt was used as a marker for Th17 cells, a nuclear hormone receptor highly expressed in Th17 cells [18]. FOXP3 was used as a marker for Tregs. It was determined that after NEC induction the number of Tregs was decreased, and Th17 cells increased. Additionally, higher levels of Th17 cells were correlated with more intestinal damage. A similar correlation was reported in a mouse model by Niño et al. [19]. They found that after NEC induction in mice Th17 cell activation was increased, as were levels of its product interleukin-17 (IL-17). Furthermore, NEC severity increased when the mice were depleted of Tregs prior to NEC induction. In this study, all-trans retinoic acid (ATRA) was given during NEC induction as experimental intervention. In the treated mice, NEC incidence and Th17 cell levels were reduced while Treg levels were preserved.

These increases in Th17 cells and IL-17 were also seen in patients with NEC. Pang et al. collected blood samples from patients with NEC and age-, sex- and weight-matched controls, and analyzed PBMCs [20]. They found that the CD4^+^ T cells of infants with NEC had increased transcription levels of RORC, a transcription factor involved in Th17 cell differentiation, and IL-17 compared to controls [21]. Additionally, these CD4^+^ T cells of patients with NEC had lower levels of FOXP3^+^ transcription, indicating decreased differentiation into Tregs. To investigate the Treg function in NEC, the same group of Pang et al. used PBMCs collected from infants displaying symptoms of NEC and age-, GA- and BW-matched infants without NEC [11]. Again, it was determined that the Treg frequency was significantly lower in infants with NEC compared to neonates without NEC. These cells also secreted fewer anti-inflammatory and Treg differentiation inducing cytokines in neonates with NEC [22,23]. In addition, the level of Th17 cells and IL-17 expression were increased in infants with NEC. Finally, it was found that in neonates with NEC, IL-17 production by CD4^+^ T cells was not reduced when Tregs were present, which did occur with cells of patients without NEC. Tregs from neonates with NEC also had lower expression of Treg associated genes.

In short, in addition to a reduced Treg level, increased levels of Th17 cells and IL-17 were seen in human neonates and animals with NEC. Additionally, the inflammatory regulation functions of Tregs were reduced in infants with NEC.

#### 3.2.3. Increased Levels of CCR9 + Tregs in NEC

Ma et al. performed a study in both mice and patients with NEC, to investigate frequency of CCR9^+^ T cells [24]. CCR9 is chemokine receptor 9, which regulates the T cell migration into the small intestine [25,26,27]. It is a rare cell type which has the characteristics of a Treg, but produces IL-17 and has reduced suppressive activity, which causes increased inflammation [24]. They found that mice with NEC had increased levels of Th17 cells and CCR9^+^ IL-17^+^ Tregs, but decreased levels of regular Tregs in the intestine. Levels of circulating CCR9^+^ IL-17^+^ Tregs were also increased. These increased CCR9^+^ IL-17^+^ Treg levels caused more severe NEC symptoms in mice. In peripheral blood of neonates with NEC, compared to GA-, birth weight (BW)- and sex-matched preterm neonates without NEC, the level of CCR9^+^ IL-17^+^ Tregs was also found to be elevated. The immunosuppression of CCR9^+^ Tregs from patients with NEC was impaired compared to control patients’ Tregs. Additionally, it was determined that neonates with NEC had increased expression of IL-6, which can promote polarization of standard Tregs towards CCR9^+^ IL-17^+^ Tregs. In a second study by the same group, Ma et al. investigated whether NEC can be treated using melatonin [28]. During NEC induction, mice were given either melatonin or saline. Additionally, some mice were given IL-17, and others were depleted of Tregs. Melatonin administration resulted in a reduction in NEC severity, mortality and morbidity. The level of Th17 cells was decreased in these mice, and Tregs were increased compared to saline-treated NEC-induced mice. The protective effect of melanin was negated after either IL-17 administration or Treg ablation. 

In conclusion, the level of inflammatory CCR9^+^ IL-17^+^ Tregs is increased in both mice and neonates with NEC, while standard inflammation regulating Treg levels are reduced.

**Table 1:** Characteristics and main findings of included T cell related studies.

Author	Year	Study Design	Species	Sample Size	NEC Induction	Methods	Histological NEC Score	Flow Cytometry	T Cell Markers	Most Relevant Findings
Huenecke [14]	2016	Case control	Human	50 total, 40 cases, preterm; 10 controls, term	No	Infants grouped based on GA. Immune cell subsets were analyzed on PB samples.	No	Yes: on blood	Treg: CD4^+^ + CD25^+^ + CD127^dim^; naive T cells: CD3^+^ + CD4^+^ or CD8^+^ + CD45RA^+^ + CD62L^+^; central memory T cells: CD3^+^ + CD4^+^ or CD8^+^ + CD45RO^+^ + CD62L^+^; effector memory T cells: CD3^+^ + CD4^+^ or CD8^+^ + CD45RO^+^ + CD62L^−^; effector memory RA T cells: CD3^+^ + CD4^+^ or CD8^+^ + CD45RA^+^ + CD62L^−^	B cell numbers were decreased in preterm infants compared to term infants at time of birth. Tregs frequency was highest in preterm infants at GA of 26 weeks, but declined during first weeks of life.
Weitkamp [15]	2009	Case study	Human	59 total, all intestinal surgical cases	No	Remnant human tissue samples of intestinal surgical cases of NEC, perinatal spontaneous intestinal perforation, and congenital intestinal obstruction. Immunohistochemistry was performed.	No	No	Treg: FOXP3^+^; effector T cells: CD3^+^ + CD4^+^ or CD8^+^	FOXP3^+^ cells were found in the large and small intestine as early as 23 weeks GA. There was no change in the ratio of FOXP3^+^ to CD4^+^ or CD8^+^ cells with postnatal exposure. CD3^+^, CD4^+^, CD8^+^ and FOXP3^+^ cells were decreased in NEC patients, but present in same ratios as the other diseases.
Weitkamp [7]	2013	Case control	Human	48 total, 18 cases, 30 controls	No	Fresh ileal tissue samples from NEC patients and non-NEC intestinal surgical patients matched for GA. LPMCs and Tregs were isolated. T cell suppression assay and PCR on total ileal RNA performed.	No	Yes: on remnant surgical intestinal tissue	Treg: CD4^+^ + Cd25^+^ + FOXP3^+^ + CD127^low^ + CD45RO; effector T cells: CD4^+^ or CD8^+^	Proportions of Tregs to CD4^+^ or CD8^+^ T cells were lower in ileum of NEC patients. Absolute Treg number was decreased in NEC patients. Treg proportions increased after NEC healing.
Dingle [6]	2013	Experimental	Sprague Dawley rat pup	134 total	Yes: formula fed, starved 12 h after birth, hypoxia	Rats were divided into groups: dam-fed; NEC induction; NEC induction + Treg supplementation; NEC induction + saline supplement; NEC induction + active Treg supplementation; NEC induction + active Teff supplementation.	Yes: terminal ileum HE staining	Yes: on spleen, thymus, mesenteric lymph node and terminal ileum	Treg: CD3^+^ + CD4^+^ + FOXP3^+^; effector T cells: CD3^+^ + CD4^+^ or CD8^+^	The Treg frequency in the ileum was significantly lower in NEC-induced rats. NEC-induced rats had lower NEC incidence after Treg transfer. Levels of ileal Tregs were higher towards normal after Treg transfer. CD25 expression and T cell maturation occurred in rats after the Teff transfer.
Egan [17]	2016	Experimental	Wild type; RAG−/−; IL-17-GFP; RORyt-GFP mice	170 total	Yes: formula fed, hypoxia, NEC patient enteric bacteria supplementation	Enterocytes incubated with LPS, IL-17A or vehicle for 6 h. ELISA and immunoblots performed.	Yes: ileum HE staining	Yes, on lamina propria cells	Treg: CD4^+^ + FOXP3^+^; Th17 cells: CD4^+^ + RORγt^+^	RORγt^+^ T cells were increased and FOXP3^+^ T cells were decreased after NEC induction compared to wild-type mice. T cell deficient (RAG−/−) mice were protected from developing NEC.
Niño [19]	2017	Experimental	Wild-type mice, enterocyte culture	120 total	Yes: formula fed, hypoxia, NEC patient enteric bacteria supplementation	Enterocytes treated with IL-17A or vehicle for 6 h. Immunohistochemistry performed. Mice were divided into groups: dam-fed; dam-fed + ATRA supplement; NEC induction; NEC induction + ATRA supplement; NEC induction + Treg depletion.	Yes: terminal ileum HE staining	Yes: on lamina propria cells	Treg: CD4^+^ + FOXP3^+^; Th17 cells: CD4^+^ + IL-17^+^	Th17 cell activation and IL-17 expression was increased in NEC mice. Treg depletion lead to increased intestinal damage after NEC induction. ATRA preserved levels of Tregs in NEC-induced mice. ATRA decreased Th17 cell induction and IL-17 expression in NEC-induced mice.
Pang [20]	2018 (June)	Case control	Human	30 total, 15 cases, 15 controls	No	Blood samples from infants displaying clinical NEC symptoms, included after surgical procedure. Non-NEC controls matched for BW, GA and sex. RT-PCR and cytokines ELISA performed.	No	Yes: on blood	Treg: CD4^+^ + FOXP3^+^; Th17 cells: CD4^+^ + RORγt^+^	Monocytes from NEC infants had higher TLR4 expression. CD4^+^ T cells of NEC patients had higher RORC and lower FOXP3 transcription than controls, and expression of IL-17 was increased in patients.
Pang [11]	2018 (October)	Case control	Human	30 total, 15 cases, 15 controls	No	Blood samples from infants displaying clinical NEC symptoms, included after surgical procedure. Non-NEC controls matched for BW, GA and sex. RT-PCR and cytokines ELISA performed.	No	Yes: on blood	Treg: CD3^+^ + CD4^+^ + CD25^+^ + FOXP3^+^; Th17 cells: CD3^+^ + CD4^+^ + IL-17^+^	Treg frequency was significantly lower in NEC patients. Tregs of NEC infants have reduced expression of Treg related genes. The frequency of IL-17^+^ CD4^+^ T cells and level of IL-17 was significantly higher in NEC infants.
Ma [24]	2019	Experimental and case control	Wild-type mice, human	Mouse: 83 total Human: 157 total, 77 cases, 80 controls	Mouse: Yes: formula fed, hypoxia, NEC patient enteric bacteria supplementation Human: No	Mouse: Mice divided into groups: dam-fed; NEC induction; NEC induction + anti-IL6R supplement. Cytokine ELISAs and total protein Western blots performed. Human: Blood samples from infants diagnosed with NEC and BW, GA and sex matched control neonates. Tregs and CCR9+ CD4+ T cells isolated. Treg polarization, T cell proliferation and suppression assays performed. RT-PCR performed on total RNA from CCR9+ CD4+ T cells.	Mouse: Yes: ileum HE staining Human: No	Mouse: Yes: on ileum Human: Yes: on blood	Mouse: Treg: CD4^+^ + FOXP3^+^; Th17 cells: CD4^+^ + IL-17^+^ Human: Treg: CD4^+^ + FOXP3^+^; Th17 cells: CD4^+^ + RORγt^+^	Mouse: Levels of Th17 cells and IL-17^+^ Tregs increased and Treg levels decreased after NEC induction compared to controls. Frequency of circulating CCR9^+^ IL-17^+^ Tregs increased after NEC inductions. Increased level of circulating CCR9^+^ IL-17^+^ Tregs correlated negatively to severity of intestinal damage. Human: Monocytes from NEC infants had higher TLR4 expression. CD4^+^ T cells of NEC patients had higher RORC and lower FOXP3 transcription than controls, and expression of IL-17 was increased in patients.
Ma [28]	2020	Experimental	Wild-type mice	60 total	Yes: Formula fed, hypoxia, cold stress	Mice divided into groups: dam-fed; NEC induction; NEC induction + melatonin supplement; NEC induction + saline supplement; NEC induction + melatonin supplement + Treg ablation; NEC induction + saline supplement + Treg ablation.	Yes: ileum HE staining	Yes: on lamina propria mononuclear cells	Treg: CD4^+^ + FOXP3^+^; Th17 cells: CD4^+^ + IL-17^+^	Levels of Th17 cells increased and levels of Tregs decreased after NEC induction. Melatonin supplementation during NEC induction normalized Th17 cell and Treg levels and decreased NEC incidence. NEC protection by melatonin was impaired after Treg ablation.

### 3.3. Immune Modulation in NEC

**Figure 3:**
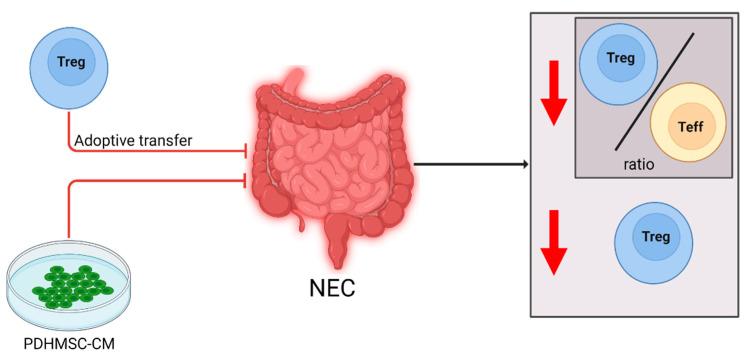
Cellular changes observed in NEC patients and experimental animal models and the effect of immune modulating interventions. After NEC diagnosis or induction, the Treg/Teff cell ratio and overall Treg levels were decreased (downward arrow). Supplementation of PDHMSC-CM negated the decrease in Tregs, improving the immune regulation, and reduced development of NEC in a rat model. Adoptive Treg-transfer reduced NEC development in a mouse model.

As described in the previous section, both animal models and patients with NEC show increased levels of Th17 cells and inflammatory cytokines and decreased levels of Tregs, as well as reduced immunosuppressive function of the Tregs. These findings imply that in NEC, the immune system is skewed towards inflammation. Multiple selected articles performed immunomodulatory experiments in newborn animals to confirm this implication. These articles will be discussed here and summarized in Figure 3 and Table 2.

Schulz et al. induced NEC in a heme oxygenase-1 (HO1) knockout mouse model [29]. It was previously shown that HO1-deficient mice are more vulnerable to NEC-like intestinal injury [30]. In mice after NEC induction, Treg frequency was significantly decreased. The intestinal NEC scores of the mice were correlated with Treg/effector T cell ratios. Furthermore, an increased Treg/effector T cell ratio appeared to be associated with attenuation of intestinal damage. After transfer of wild-type thymic or splenic Tregs during NEC induction intestinal damage scores and incidence of NEC decreased. 

Chen et al. transferred medium of bone marrow-derived mesenchymal stem cells (BM-MSCs) with propyl hydroxylase 2 (PHD2) silenced into an experimental NEC rat model [31]. A previous study showed that the conditioned medium secreted by the cultured MSCs can help repair intestinal injury [32]. PHD2 is a protein which regulates two transcription factors involved in paracrine activity of the cell [33]. Chen et al. investigated how the conditioned medium of PHD2 silenced BM-MSCs (PHDMSC-CM) affects experimental NEC [31]. As in all previously discussed articles, the rats had a decreased level of FOXP3^+^ Tregs in the mesenteric lymph nodes after NEC induction when compared to controls. Treatment with PHDMSC-CM reduced NEC incidence compared to NEC induction only, and intestinal damage was reduced. The PHDMSC-CM-treated rats had a significantly increased number of FOXP3^+^ Tregs in the mesenteric lymph nodes compared to NEC induction-only pups.

**Table 2:** Characteristics and main findings of included inflammation related studies.

Author	Year	Study Design	Species	Sample Size	NEC Induction	Methods	Histological NEC Score	Flow Cytometry	T Cell Markers	Most Relevant Findings
Schulz [29]	2015	Experimental	Wild-type mice: HO1−/−; HO1+/−	51 total	Yes: formula fed, hypoxia	HO1+/− mice were injected with wild-type Tregs.	Yes: intestine HE staining	Yes, on ileum	Treg: CD4^+^ + CD25^+^ + FOXP3^+^; effector T cells: CD4^+^	Treg frequency was decreased in NEC induced mice. High Treg/effector T cell ratio was associated with attenuated intestinal damage. Treg transfer during NEC induction reduced intestinal damage scores and NEC incidence.
Chen [31]	2020	Experimental	Sprague Dawley rats	58 total	Yes: formula fed, starved 12 h after birth, hypoxia	BM-MSCs transfected with PHD2 or GFP silencing lentivirus. Cytokine assay on conditioned medium. Conditioned medium was supplied to rats. Cytokine arrays, qPCR and Western blot performed.	Yes: terminal ileum HE staining	Yes, on enterocyte culture and rat mesenteric lymph nodes	Treg: CD4^+^ + FOXP3^+^	PHD2 knockdown reduced NEC incidence and ileal damage. PHD2 knockdown increased number of FOXP3 Tregs in the mesenteric lymph nodes. PHD2 knockdown BM-MSCs produced more anti-inflammatory and Treg inducing cytokines.

### 3.4. The Role of Milk on NEC and Tregs 

**Figure 4:**
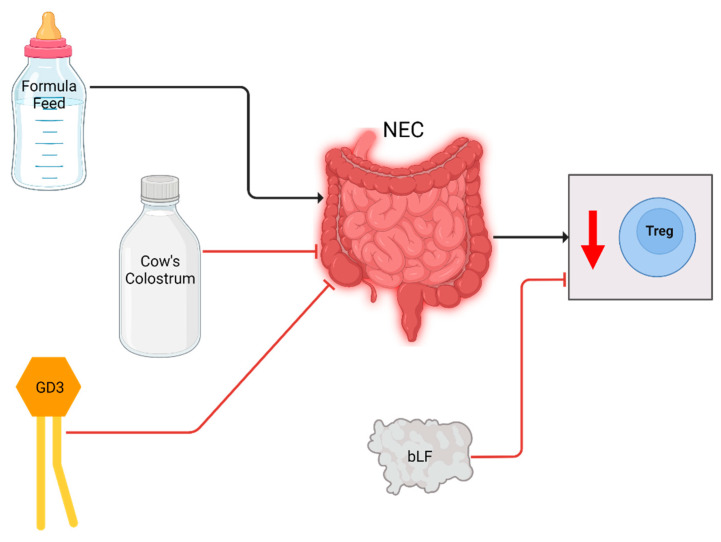
Cellular changes observed in NEC patients and experimental animal models and the effect of breastmilk related interventions. After NEC diagnosis or induction, Treg levels were decreased (downward arrow). Formula increased NEC incidence and decreased Treg levels in piglets, whereas colostrum reduced NEC incidence and normalized Treg levels. GD3 supplementation negated the decrease in Tregs in a rat model. bLF supplementation increased Treg levels of individual NEC patients.

If breastmilk is insufficiently available, infant formula is often used in hospitals. However, formula lacks multiple components of maternal milk, which are beneficial to the immune system [34,35,36]. Preterm formula feeding is shown to cause damage in the intestine, and is associated with NEC development [37]. Because of this, several studies were performed to determine the influence of colostrum or specific components of maternal milk on NEC development and progression. The results have been visualized in Figure 4 and Table 3.

Li et al. used preterm pigs as a model for preterm infants to assess the influence of formula versus colostrum feeding on NEC development [38]. Cow’s colostrum could be used as an alternative to formula feeding, as it contains components similar to maternal milk [39]. Li et al. found that frequency of Tregs was regulated by the type of feed in the first 4 days of life. In pigs fed cow’s colostrum in the first 4 days, no matter what they were fed in the days after, the Treg frequency was higher compared to pigs that were fed with formula feeds during the entire 9 days. The increased Treg frequency coincided with decreased intestinal damage.

The group of Xu et al. used dietary ganglioside 2-galactosylglucosyl ceramide (GD3) to reduce NEC incidence and severity in a rat pup model [40]. Gangliosides have been found in maternal milk, and have been seen to reduce inflammation [41,42]. They determined that after NEC induction the intestinal damage was significantly increased and the FOXP3 production was reduced compared to control rats. In rats treated with GD3 during NEC induction, the ileum showed less damage compared to NEC only pups, and FOXP3 expression was increased compared to both NEC only and control pups.

Akin et al. investigated the effect of bovine lactoferrin (bLF) on NEC development in either very low birth weight (VLBW) neonates or infants born before 32 weeks GA [43]. LF is the major whey protein in mammalian milk, and has anti-inflammatory and enterocytic proliferative properties [44]. This group investigated the T cell subsets present in blood at time of birth and discharge. NEC developed in the untreated group only. However, no significant differences between Treg levels of bLF-treated and -untreated groups, or between patients with or without NEC were found, possibly due to small sample size. In bLF-treated infants individually, Treg level did increase significantly between birth and discharge from the hospital.

**Table 3:** Characteristics and main findings of included breastmilk related studies.

Author	Year	Study Design	Species	Sample Size	NEC Induction	Methods	Histological NEC Score	Flow Cytometry	T Cell Markers	Most Relevant Findings
Li [38]	2020	Experimental	Preterm piglets	74 total	No	Piglets divided in groups: 4 day colostrum fed; 4 days formula fed. After day 4, piglets again divided in groups: continuation of previous diet until day 9; switched diet until day 9; euthanasia. Cytokine ELISAs performed before euthanasia. qPCR performed for mucosal gene expression.	Yes: proximal, middle and distal smallintestine HE staining	Yes: on arterial blood	Treg: CD4^+^ + CD25^+^ + FOXP3^+^; effector T cells: CD3^+^ + CD4^+^ or CD8^+^	Pigs fed colostrum for 4 days had lower NEC incidence and less severe intestinal lesions. After 9 days, NEC incidences were similar among all the different groups. Treg frequency was higher in groups fed colostrum at any point compared to pigs fed formula only.
Xu [40]	2013	Experimental	Sprague Dawley rats	90 total	Yes: formula fed, hypoxia, cold stress	Rats divided into groups: dam-fed; NEC induction; NEC induction + GD3 supplement. Cytokine array and FOXP3 immunoblot performed. FOXP3 immunofluorescence performed on ileum.	Yes: distal ileum HE staining	No	Treg: FOXP3^+^	GD3 supplementation reduced NEC incidence and severity scores. Levels of inflammatory cytokines were increased in NEC rats, but normalized after GD3 supplementation. FOXP3 expression was lower in the lamina propria of NEC rats. FOXP3 expression increased after GD3 supplementation.
Akin [43]	2014	Randomised clinical trial	Human	50 total, 25 lactoferrin prophylaxis, 25 placebo	No	Neonates with VLBM or GA less than 32 randomised to bLF or placebo groups. bLF and placebo supplemented in milk or formula once daily for entire hospital stay. Blood samples obtained at birth and discharge.	No	Yes: on blood	Treg: CD4^+^ + CD25^+^ + FOXP3^+^	In control patients, 20% of neonates developed NEC. In the bLF-treated group, none of the neonates developed NEC. No significant differences were found in Treg levels between groups. Level of Tregs increased significantly between birth and discharge in bLF-treated infants.

### 3.5. The Effect of Probiotics on Tregs

**Figure 5:**
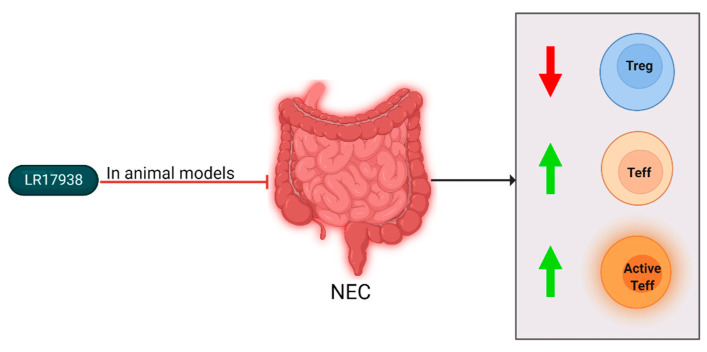
Cellular changes observed in NEC patients and experimental animal models and the effect of LR17938 supplementation. After NEC diagnosis or induction, Treg levels decreased (downward arrow), and Teff levels and activation increased (upward arrow). In mouse and rat models, LR17938 supplementation reduced NEC incidence. In a mouse model, the LR17938 supplementation negated the Treg level reduction. In neonates with NEC, LR17938 supplementation did not show effects.

Finally, probiotics have been shown to influence the inflammatory processes mediated by TLRs to increase tolerance against pathogens [45]. The effects are, however, strain specific and not generalized for all probiotics [46,47,48,49,50,51]. In the studies selected in this review, Lactobacillus reuteri DSM 17938 (LR17938) was supplemented in formula feeding. LR17938 was derived from a bacterium found in human breast milk, and was adapted to remove antibiotic resistance genes [52]. Findings of the selected articles have been summarized in Figure 5 and Table 4.

The group of Liu et al. studied the effect of LR179368 supplementation on T cell subsets in experimental NEC [5]. They found that in control rats Treg frequency increased rapidly during first days of life. When the Treg levels of control rats were compared to NEC-induced rats, it was found that at all measured points of time the frequency was lower in rats with NEC. After LR17938 supplementation, the Treg levels in the ileum of rats with NEC were significantly increased compared to untreated NEC-induced rats. The same group also studied LR179368’s effect in mice [53]. In the NEC-induced mice, the levels of effector memory T (Tem) cells were increased in the intestines, and Treg levels were decreased. LR17938 supplementation normalized Tem and Treg levels, and also resulted in reduced histological NEC incidence and severity. Hoang et al. studied the mechanism by which LR17938 induces its anti-inflammatory effects [54]. It was found that in mice with NEC, the level of activated effector T cells (Teff) was increased. Additionally, the percentage of Tregs was decreased in NEC-induced mice. As with Liu et al., these levels normalized after LR17938 feed supplementation.

Qazi et al. used LR17938 as feeding supplementation for extremely low birth weight (ELBW) or extremely low gestational age neonates (ELGAN), and analyzed T cell subpopulation and clinical outcomes [55]. ELBW neonates are classified as having a birth weight of less than 1000 g, and ELGAN are neonates born before 28 weeks GA. In the ELBW and ELGAN infants, it was found that percentages of total and viable lymphocytes, Helios level in Tregs, and Treg gut-homing capacity were significantly lower compared to full-term infants at 14 DOL. In ELGAN/ELBW infants who developed NEC, the levels of Tregs were very low according to authors. However, since these data were not shown in the article, there is no way to conclude whether this is significant. LR17938 supplementation had no significant effect on the restoration of T cell proportions.

**Table 4:** Characteristics and main findings of the included LR19738-related studies.

Author	Year	Study Design	Species	Sample Size	NEC Induction	Methods	Histological NEC Score	Flow Cytometry	T Cell Markers	Most Relevant Findings
Liu [5]	2013	Experimental	Sprague Dawley rats	180 total	Yes: formula fed, starved 12 h after birth, hypoxia	Rats divided into groups: dam-fed; dam-fed + LR17938 supplement; NEC induction; NEC induction + LR17938 supplement. CD3 immunohistochemistry performed.	Yes: terminal ileum HE staining	Yes: on spleen, thymus and mesenteric lymph node	Treg: CD3^+^ + CD4^+^ + FOXP3^+^; effector T cells: CD3^+^ + CD4^+^ or CD8^+^	Treg percentage increased during first days of life in dam-fed rats. Treg percentages were significantly lower in NEC rats. Treg percentage and survival rates were higher in NEC rats after LR17938 supplementation.
Liu [53]	2014	Experimental	Wild-type mice	99 total	Yes: formula fed, 12 h starvation, hypoxia, cold stress	Mice divided into groups: dam-fed; dam-fed + LR17938 supplement; NEC induction; NEC induction + LR17938 supplement.	Yes: intestine HE staining	Yes: on ileum	Treg: CD4^+^ + FOXP3^+^; activated effector T cells: CD4^+^ + CD44^+^ + CD4RB^lo^	Treg frequency decreased and effector T cell frequency increased in ileum and mesenteric lymph nodes of NEC mice. Treg and effector T cell levels remained the same as controls after LR17938 treatment.
Hoang [54]	2018	Experimental	Mice: wild type; TLR−/−	106 total	Yes, formula fed, hypoxia, cold stress	Mice divided into groups: dam-fed; NEC induction; NEC induction + anti-IL6R supplement. Cytokine ELISAs and total protein Western blots performed.	Yes: ileum HE staining	Yes: on ileum	Treg: CD4^+^ + FOXP3^+^; Th17 cells: CD4^+^ + IL-17^+^	Levels of Th17 cells and IL-17^+^ Tregs increased and Treg levels decreased after NEC induction compared to controls. Frequency of circulating CCR9^+^ IL-17^+^ Tregs increased after NEC induction. Increased level of circulating CCR9^+^ IL-17^+^ Tregs correlated negatively to severity of intestinal damage.
Qazi [55]	2020	Randomised control trial	Human	163 total 134 ELBW, 29 term.	No	Neonates with ELBW randomised into LR17938 or placebo supplement groups. Blood taken at day 14, day 28 at PMW 36 + 0. PBMC isolated.	No	Yes: on blood	Treg: CD4^+^ + CD25^+^ + FOXP3^+^ + CD127^+^; effector T cells: CD4^+^ or CD8^+^	Percentages of total and viable lymphocytes were significantly lower in ELBW neonates at 14 DOL. ELBW infants have a reduced Tbet/GATA3 ratio and Helios level in Tregs at both 14 and 28 DOL. LR17938 supplementation had no impact on the T cell proportions.

## 4. Discussion

In this scoping review, we aimed to determine whether the number of Tregs have predictive value for NEC development, and by what mechanism the Tregs influence the pathophysiology of NEC. From the literature search, 20 articles were selected which discussed Tregs in NEC in different species.

From the articles reviewed, we can conclude that in NEC patients and animals, the ratio of Tregs to Teff cells is decreased compared to infants or animals without NEC [7,11,15]. In both mouse and rat models, a decreased absolute number of Tregs is seen after induction of NEC as well [5,6,17,19,24,29,53,54]. Additionally, in both human neonates and NEC-induced mouse models, an increased level of IL-17 producing T cells and IL-17 expression was found [11,19,20,24,28]. When supplementing Tregs during NEC induction in a mouse model, the incidence of NEC is significantly reduced [29]. This would indicate that increased immune regulation during the development of NEC is beneficial, and that Tregs have a role in the development of NEC.

As mentioned before, the Treg/Teff ratio is decreased both in naturally occurring and induced NEC, and levels of the inflammatory cytokine IL-17 are increased. Additionally, adoptive transfer of Tregs can attenuate induced NEC. This indicates that a shift towards inflammation and reduced regulation have a role to play in NEC development or progression. The question that remains is at which time during the progression of NEC the decreased level of Tregs occurs. All reviewed studies discuss T cell composition either after clinical NEC diagnosis or induction of NEC. Therefore, the lower Treg ratio could occur both early during the progression of NEC and have a causative role in the pathophysiology, or at in a late stage of NEC and only be a result of the NEC progression. In that case, lower Treg levels would be a bystander effect in NEC development. One study by Huenecke et al. did try to review T cell ontogeny in preterm neonates prior to NEC development, but found no significant difference between these groups, possibly due to the small sample size [14]. This gap in knowledge on NEC development does provide interesting possibilities for future research, of which we will provide multiple examples in the next paragraph. In addition to increasing knowledge on the step by step progression of NEC, it would also be valuable to gain knowledge on the similarities and differences between naturally occurring NEC and induced NEC. Since NEC was induced in most of the reviewed articles using animal models, we were wondering whether the changes reported in NEC-induced animals would also occur in natural NEC, or if the induction process causes some alterations in the pathophysiology. More insight on this front would be advantageous for the interpretation of NEC related studies performed using animal models.

With regard to our first question, whether the number of Treg is predictive for the development of NEC, we did not find any studies which investigated this specific question. As mentioned previously, a decreased level of Tregs is found in NEC, but since the pathophysiological timeline has not been elucidated, it is not possible to conclude that this reduced level occurs prior to NEC development. We therefore think it would be relevant for future research to investigate the levels of Tregs in blood of neonates prior to NEC development, and follow these patients up to determine NEC occurrence. Additionally, future research focusing on the progression of levels of Tregs and other inflammation related cells throughout the first days of life would be informative. Studies researching T cell ontogeny in larger cohorts of preterm infants or infants with NEC could also be relevant. Finally, more research into the NEC induction process used in the animal models will increase the translation of their results to the human situation. By increasing knowledge on pathophysiology and immune cell ontogeny in NEC, more robust conclusions about the disease can be drawn in the future. 

The strengths of this scoping review are that we used stringent and transparent methods of data collection and synthesis based on the PRISMA guidelines, used multiple databases for article identification, and that at least two reviewers were involved in the selection of all articles [12]. Potential limits of this study may be that articles potentially are missed by the choice of terms and language selection. There was also a large heterogeneity within study design, complicating the drawing of conclusions. Additionally, there were some disputes based on inclusion of articles, which had to be solved by a third researcher.

In conclusion, our overview of the current literature regarding the role of Treg in the development of NEC shows that the number of Tregs is lower in animals and neonates with NEC. When utilizing experimental interventions during NEC induction, Treg frequency does not decline as seen in NEC. The question remains whether this effect is a factor on the causal pathway of NEC development or a bystander effect. No studies were found that investigated the number of Tregs as an early predictive marker of NEC development. To fill this gap in knowledge, future research focusing on the pathophysiological timeline of NEC and the involvement of regulatory T cells would be valuable for the understanding of the immunological component of the pathophysiology behind NEC.
